# Patients’ views on interactions with practitioners for type 2 diabetes: a longitudinal qualitative study in primary care over 10 years

**DOI:** 10.3399/bjgp17X693917

**Published:** 2017-12-05

**Authors:** Hajira Dambha-Miller, Barbora Silarova, Greg Irving, Ann Louise Kinmonth, Simon J Griffin

**Affiliations:** MRC Epidemiology Unit, University of Cambridge School of Clinical Medicine, Cambridge.; MRC Epidemiology Unit, University of Cambridge School of Clinical Medicine, Cambridge.; Primary Care Unit, Department of Public Health and Primary Care, University of Cambridge, Cambridge.; Primary Care Unit, Department of Public Health and Primary Care, University of Cambridge, Cambridge.; Primary Care Unit, Department of Public Health and Primary Care, University of Cambridge, Cambridge.

**Keywords:** consultation, patient–practitioner interactions, primary care, type 2 diabetes

## Abstract

**Background:**

It has been suggested that interactions between patients and practitioners in primary care have the potential to delay progression of complications in type 2 diabetes. However, as primary care faces greater pressures, patient experiences of patient–practitioner interactions might be changing.

**Aim:**

To explore the views of patients with type 2 diabetes on factors that are of significance to them in patient–practitioner interactions in primary care after diagnosis, and over the last 10 years of living with the disease.

**Design and setting:**

A longitudinal qualitative analysis over 10 years in UK primary care.

**Method:**

The study was part of a qualitative and quantitative examination of patient experience within the existing ADDITION-Cambridge and ADDITION-Plus trials from 2002 to 2016. The researchers conducted a qualitative descriptive analysis of free-text comments to an open-ended question within the CARE measure questionnaire at 1 and 10 years after diagnosis with diabetes. Data were analysed cross-sectionally at each time point, and at an individual level moving both backwards and forwards between time points to describe emergent topics.

**Results:**

At the 1-year follow-up, 311 out of 1106 (28%) participants had commented; 101 out of 380 (27%) participants commented at 10-year follow-up; and 46 participants commented at both times. Comments on preferences for face-to-face contact, more time with practitioners, and relational continuity of care were more common over time.

**Conclusion:**

This study highlights issues related to the wider context of interactions between patients and practitioners in the healthcare system over the last 10 years since diagnosis. Paradoxically, these same aspects of care that are valued over time from diagnosis are also increasingly unprotected in UK primary care.

## INTRODUCTION

It is estimated that 4 million people in the UK are living with type 2 diabetes, and there are a further 500 000 who are currently undiagnosed.^1^ This common condition accounts for significant morbidity and 10% of all premature mortality in England.^2^ Multifactorial management during the course of the disease has been shown to be effective in improving diabetes outcomes; however, the overall burden of disease remains high.^3^^,^^4^ Additional strategies for optimising management are, therefore, required. Patient–practitioner interactions refer to the relationship between practitioner and patient in the context of the consultation. This includes communication, empathy, interpersonal skills, listening, and mutual decision-making.^5^ These interactions provide an opportunity for practitioners to positively influence healthcare decisions, encourage self-management or enablement, and support patients with long-term health changes during the long course of diabetes.^6^^–^9 Previous evidence suggests that better patient experiences with practitioners are associated with lower cardiovascular (CV) risk factors such as blood pressure, glycated haemoglobin (HbA1c), and cholesterol, which in turn could lead to a delay in diabetes progression.^10^^–^14

However, there is a relative paucity of published data in UK primary care that examines patient–practitioner interactions in type 2 diabetes among participants who have been followed up over an extended period as their disease progresses and complications develop.^6^^,^^15^^,^^16^ Most previous studies have tended to utilise patient-completed quantitative questionnaires.^11^^,^^17^^,^^18^ Patients often provide high feedback scores concerning interactions and experiences of health care, based on these questionnaires. Such instruments usually contain closed or leading questions that may not adequately capture underlying patient views.^19^^–^21 Qualitative methods may, therefore, be more suited for capturing the nuances of the patient–practitioner interactions and understanding preference changes over time.^22^^,^^23^ However, only a few previous longitudinal qualitative studies have been conducted and these have not included long follow-up periods, have not specifically examined factors significant to patients over time from diagnosis, and may not be applicable to the primary care context today.^24^^–^26 Patient–practitioner interactions must be considered in the context of current UK primary care where pressures are increasing owing to a demand on practitioner consulting time, limited resources, and a growing burden of chronic diseases. As primary care adapts, it is unclear which elements of interactions with practitioners are most valued by patients after diagnosis and over time, and which could potentially be incorporated into new primary care systems and structures. This could be helpful knowledge in order to meet patient preferences, improve patient engagement, and so improve long-term health outcomes.^10^^,^^11^ Accordingly, the authors conducted a longitudinal qualitative study using free-text comment data from a patient–practitioner interaction questionnaire within existing trials. Here, participants were followed up at 1 and 10 years after diagnosis with type 2 diabetes between 2002 and 2016.^27^^–^29 This study aims to explore patient views on factors within patient–practitioner interactions that are of significance to them after diagnosis, and over a 10-year experience of living with the disease.

**How this fits in**

As primary care pressures increase and the health system adapts to these changes, patients’ experiences of patient–practitioner interactions might be impacted. To address this, a longitudinal qualitative study was conducted over 10 years, within existing trials, to explore views of patients with type 2 diabetes on factors that are of significance to them with regard to patient–practitioner interactions. Participants identified face-to-face contact with practitioners, the length of time with them, and relational continuity of care as important aspects of patient–practitioner interactions. This is concerning as it is these same aspects of care that are increasingly threatened in the current UK health system, and which may lapse with time from diagnosis.

## METHOD

The present study is based on the data collected within the existing ADDITION-Cambridge and ADDITION-Plus trials between 2002 and 2016. A detailed description of the study designs and rationale has been previously reported.^27^^,^^28^ In short, ADDITION-Cambridge was a cluster randomised controlled trial in which patients were screened for type 2 diabetes followed by either routine care (control group) or intensive multifactorial therapy (intervention group). The ADDITION-Plus trial was then nested within the treatment group of the ADDITION-Cambridge study to examine the efficacy of an additional contribution of a facilitator-led behaviour change intervention. The inclusion criteria for the trials were individuals registered in one of the participating 34 general practices across East Anglia, and aged 40–69 years. Exclusion criteria were females who were pregnant or lactating, participants with psychiatric illness, or those with a likely survival prognosis of <1 year. In total, 1106 individuals agreed to participate: those who had been diagnosed with type 2 diabetes following screening in the ADDITION-Cambridge study (*n* = 867); and those who were clinically diagnosed during the previous 3 years in participating general practices in ADDITION-Plus (*n* = 239).

Participants completed the CARE measure tool about their experiences of patient–practitioner interactions 1 and 10 years after recent diagnosis with type 2 diabetes, describing actual methods of the trial.^18^^,^^30^ The CARE measure tool is a patient-rated experience measure that has been developed and undergone validation within the primary care setting and has been used by doctors, allied health professionals, and nurses. The measure, thus, appropriately reflects the range of practitioners that interact with patients in the management of type 2 diabetes in primary care. The CARE measure tool includes 10 questions based on a Likert scale ranging from one to five. The scoring system for each item is: 1 = ‘poor’, 2 = ‘fair’, 3 = ‘good’, 4 = ‘very good’, and 5 = ‘excellent’. All 10 items are then added together, giving a maximum possible score of 50, and a minimum of 10. Participants consistently rated patient–practitioner interactions as high on the CARE scores and there was little variation in CARE score at both time points; mean (SD) at 1-year follow-up was 39 (9), and at 10 year follow-up was 39 (10). A description of the quantitative results from the CARE measure has been published previously.^14^

### Qualitative component

A single open-ended question at the end of the CARE measure asks: *‘If you would like to add further comments, please do so here.’*^29^ These responses were anonymised, collated, and imported into NVivo 10 to aid a qualitative descriptive analysis.^31^ The free-text comments were often brief, precluding detailed thematic analysis to understand relationships between themes. Therefore, a descriptive approach to analysis was more appropriate.^32^^,^^33^ Two researchers (a psychologist and a UK academic GP) repeatedly read comments to achieve textual familiarisation, followed by comparison of transcripts. A third researcher (a UK academic GP) independently reviewed 10% of the transcripts to ensure validity of coding. The coding was guided pragmatically by our research aim to capture underlying views on patient–practitioner interactions that may not be reflected within the high quantitative CARE scores. However, allowances were made for inductive analysis of unanticipated topics. An iterative process of open coding was initially used, in which short descriptive codes were applied to each comment. Codes were reviewed, compared to identify similarities and differences, and then grouped into higher-level categories to provide a descriptive summary of the comments.

Data were analysed cross-sectionally (at each time point) first, and then moved both backwards and forwards between time points across individuals to describe emergent topics and variations. To support the validity of emerging findings, an interim descriptive account was discussed with the research team early in the analysis stage. Analysis was continued until theoretical saturation was reached and no new topics emerged. The study team met regularly to discuss findings and negative cases, and to resolve discrepancies in descriptions with a view to ensure rigour in analysis.^34^^,^^35^ Finally, the process and topics were discussed with a peer debriefer of the same department as the researchers, who corroborated the findings, strengthened the analysis and ensured trustworthiness of the process.

## RESULTS

Out of 1106 participants who completed the CARE questionnaire at 1-year follow-up, 311 included a comment (28% of questionnaire respondents), 101 of 380 participants who completed the questionnaire at 10-year follow-up also provided a comment (27% of questionnaire respondents), and 46 of the same participants responded at both time points. The characteristics of the participants who provided a comment are shown in Table 1. Participants’ comments varied in length and depth of information provided. A summary of participants’ views on patient–practitioner interactions at 1 year and 10 years after diagnosis are presented in this article. The illustrative quotations are identified by study number, where 1 indicates 1-year follow up and 10 indicates 10-year follow-up. Quotations are divided into three main topics: face-to-face contact with practitioners; length of time with them; and relational continuity of care. These topics were identified both within the cross-sectional analysis and in the analysis of the 46 cases that responded at both time points.

**Table 1.** Participant characteristics

**Characteristic**	**1-year time point**	**10-year time point**
Participants who included free-text comment,^a^ *N*	311	101
Sex (male), *n* (%)	196 (63)	53 (53)
Age, years, mean (SD)	60 (6)	72 (7)
White ethnic group, *n* (%)	299 (96)	100 (99)
Employed, *n* (%)	230 (74)	32 (32)
HbA1c, %, mean (SD)	6.7 (0.9)	7.2 (1.3)
Systolic blood pressure, mmHg, mean (SD)	136 (19)	133 (13)
Diastolic blood pressure, mmHg, mean (SD)	79 (10)	73 (8)
Body mass index, kg/m^2^, mean (SD)	32 (5)	29 (4)
CARE score,^b^ mean (SD)	39 (9)	39 (10)

a Participants included free-text comment in CARE questionnaire.

b Consultation and relational empathy measure. SD = standard deviation.

### Face-to-face contact with practitioner

Early in the course of type 2 diabetes, a large number of participants described the frequency of face-to-face contact with their practitioner (including doctor, nurse, healthcare assistant and dietician). There were often comments about the regularity of face-to-face interactions that seemed to be received positively:

‘Dr X is my GP and I see him each time for my diabetes check-ups — he gives me all the time I need–—is never in a rush to get me out of the surgery and discusses everything with me — he also explained all the various readings that have been taken from my blood tests — he is excellent.’

(Patient 591-1)

‘On a regular basis visit to X surgery attended by nurse Y who looks after me and explains all my medications and various tests — blood — height — weight — blood pressure — cholesterol and test feet; Also regular visits to chiropodist and regular eye test and new glasses now vision is perfect — also regular urine sample test.’

(Patient 575-1)

‘See diabetic nurse every 3 months with urine sample, blood pressure tested and weighed.’

(Patient 780-1)

‘GP seen on yearly checks.’

(Patient 614-1)

‘Discussion are on a regular per mensum routine with the practice nurse.’

(Patient 627-1)

‘My GP and practice nurse make time and ensure that issues raised are fully covered.’

(Patient 750-1)

‘Regular checks have always been with the practitioner nurse.’

(Patient 805-1)

‘Regular contact with the practice nurse.’

(Patient 909-1)

Later in the course of diabetes at 10-year follow-up, a large number of comments that were brief, and referred to the fact that participants had not experienced any recent patient–practitioner interactions, were observed. Repeated reading of comments suggested that face-to-face contact with a practitioner had been absent from anywhere between 10 months to 5 years. A few participants related this to difficulties with obtaining appointments:

‘I have had no contact with the doctor for 5 years.’

(Patient 325-10)

‘I have never seen a doctor only a nurse.’

(Patient 327-10)

‘I have not seen a doctor for several years.’

(Patient 329-10)

‘I have not seen my doctor in the last 3 months.’

(Patient 310-10)

‘I have NOT seen the diabetic nurse for over 6 months in spite of repeated attempts. That is why I have changed surgeries.’

(Patient 333-10)

‘Not seen doctor for diabetes for a long time.’

(Patient 661-10)

‘Have had no contact with the doctor for 5 years.’

(Patient 359-10)

‘I have not seen my doctor for a long while (>1year).’

(Patient 375-10)

‘The system of getting the appointment in an advanced country like UK is not acceptable, in the current system, there is no certainty of seeing the doctor. In the current system, Mon–Fri ring the surgery @ 8.30 and ask for the appointment, most of the time telephone line is engaged, sometime you keep on ringing for 45mins, when you do get through to receptionist, you ask for the appointment the receptionist will say sorry we are full today ring tomorrow. There is no guarantee for tomorrow appointment.’

(Patient 376-10)

### Length of patient–practitioner interaction

Through the course of the 10-year study, participants made frequent references to inadequate time being available during interactions with practitioners. Participants described short and rushed patient–practitioner interactions at both time points, but comments were more frequent at 10-year follow-up:

‘I have felt I need more information; the nurses are always busy and don’t have enough time.’

(Patient 16-1)

‘My doctor does not appear to have the time to explain or discuss things with me.’

(Patient 33-1)

‘The visits are rushed affairs.’

(Patient 246-1)

‘I do not consider 5 minute visit is long enough to make all the valued opinions in 12 months.’

(Patient 996-1)

‘It’s a bit like a conveyor belt.’

(Patient 1022-10)

’Frequently unable to address all health issues because “ran out of time” (i.e. 10 minutes) … make another appointment and so it goes on.’

(Patient 311-10)

‘They are under time pressure.’

(Patient 378-10)

‘She is a very nice person — I like her — but she is too busy.’

(Patient 1044-10)

### Continuity of care

Issues around continuity of care seemed to recur frequently in the data at 1- and 10-year follow-up. Many participants seemed to place great importance on relational continuity of care that was distinct from comments on face-to-face contact:

‘Not always guaranteed to see the same person.’

(Patient 275-1)

‘Only problem is as the practice is large, one does not always see the same doctor each time.’

(Patient 279-1)

‘Having to see a different nurse on each visit makes things difficult.’

(Patient 246-1)

‘Seeing the same person when I go to the GP is very helpful. Also, having 3 month checks keep me on track.’

(Patient 372-1)

‘I have discussed my problem with more than one doctor in the practice. I would be happier to deal with one all the time.’

(Patient 375-1)

‘Change of GP too frequent to fully answer each heading.’

(Patient 294-10)

‘I’ve never seen a doctor for my diabetes, only nurses which are forever changing. So, I mainly look after myself.’

(Patient 315-10)

‘No fault of the doctors but have not seen the same doctor on a regular basis.’

(Patient 317-10)

‘I have had seven GPs since I joined this practice. They come and go with regular monotony. The last one left before Christmas.’

(Patient 452-10)

## DISCUSSION

### Summary

The analysis of free-text comments aimed to provide patient views on patient–practitioner interactions that may not have been captured within the quantitative questionnaire, and to explore these after diagnosis and after 10 years of living with the disease. This approach identified the significance that patients placed on face-to-face contact, length of interactions with practitioners, and relational continuity of care. Comparing the early responses with those after 10 years suggests that patients continue to value these factors but find delivery less satisfactory over time. Rather than the interpersonal skills or attributes of patients or practitioners within the consultation, which patients scored highly in the quantitative questionnaires, the study illuminates aspects related to the wider context of health services within which these interactions occur. Health service re-organisation may not enable the patient preferences identified in these analyses. Preferences were met less over time as diabetes duration increased, and may be thwarted further by the current UK primary care system. This is owing to the growing clinical workload, increased prevalence of chronic diseases, declining workforce, and inadequate resourcing in primary care.^36^

### Strengths and limitations

A main strength of this study is the extended longitudinal follow-up from recent diagnosis to 10 years of living with the disease. Ten-year follow-up data were collected right to the end of 2016, which makes this study relevant to primary care today. The inclusion of free-text comments also complements the researchers’ previous quantitative results by providing participants with the opportunity to expand on topics of priority to them.^14^ These aspects of patient–practitioner interactions raised by patients were not previously captured within the quantitative questionnaire components on patient–practitioner interaction; thus, the qualitative components have enriched the authors’ understanding. Further strengths include the robustness of qualitative analysis, which included item checking across 46 respondents at both time points, regular discussions within the team, interim accounts, peer debriefing, negative case analysis, and continued analysis until no new topics emerged for sufficient data saturation to be achieved at both time points.

Limitations included not identifying individuals for triangulation of findings and inability to perform member-checking. Response rates were low at both sampling points, and follow-up was a limitation given the duration of the study. However, the characteristics of those who did comment at both time points did generally reflect patients with type 2 diabetes within practices included in this study. Participants were heterogeneous in relation to ages, rates of complications, morbidity, and mortality, which may itself have contributed to the diminishing follow-up over the 10 years. Although the study succeeded in obtaining a diverse sample of participants in these respects, the study sample mainly included white males. Over the course of the study participants were additionally likely to have seen multiple practitioners, and no detailed information about the number of contacts was obtained, other than that provided by the participants themselves. There was, therefore, uncertainty about specific interactions that participants may be referring to, or their context. The present study was set within existing trials and it is, therefore, possible that the trial setting and the intervention itself could have influenced patients’ perceptions of care. Furthermore, as the trial intervention did pursue a person-centred approach, it is plausible that the study findings may under-report the experiences of the average patient with diabetes outside the trial. Finally, some of the comments were short, which restricted the ability to provide in-depth and detailed interpretation of the data. Work towards a qualitative interview study within this population is already underway that will attempt to probe participants for more detailed and elaborate views on patient–practitioner interactions over the course of type 2 diabetes.

### Comparison with existing literature

The findings are consistent with previous evidence, including a recent article by Burt and colleagues*,* which demonstrates the value of qualitative research in unmasking important patient experiences that may not be captured in quantitative questionnaires.^21^ This qualitative study is also consistent with previous evidence in highlighting the value that patients with type 2 diabetes,^6^^,^^16^^,^^37^^–^39 and other chronic diseases more widely, place on practitioner continuity, length of time of, and in-person contact.^40^^,^^41^ Few such qualitative studies have included longitudinal follow-up of newly diagnosed patients with diabetes and none has specifically examined patient–practitioner interactions.^6^^,^^37^^,^^42^ However, these studies do highlight patient priorities in terms of these interactions. These aspects are also valued by practitioners. A recent survey of 16 000 GPs found that 90% reported current consultation lengths as inadequate, and 80% highlighted the value of continuity in optimal health care.^43^

The findings reported here are also consistent with recent quantitative evidence on the delivery of primary care in the UK that highlights potential organisational-level barriers to optimal patient–practitioner interactions. In particular, one study of over 100 million consultations between 2007 and 2014 in primary care reported a significant increase in GP workloads that are reaching maximum capacity in the number and length of interactions with practitioners.^44^ Large increases in telephone call interactions were also reported over this time, which is contrary to the face-to-face contact that the researchers in this study found was preferable to patients. A decrease in the number of full-time equivalent GPs per 100 000 patients was additionally reported, which corroborates our participant concerns about reduced relational continuity of care. Although these aspects of patient–practitioner interactions are valued by patients, there is much debate about whether they are associated with improved outcomes, with mixed findings from previous studies.^45^^–^48 However, most previous research tends to focus on each of these aspects in isolation and there is a paucity of evidence on their combined impact on processes such as patient engagement, behaviour change, and on long-term outcomes in type 2 diabetes, or chronic diseases more widely.

Additionally, most trial evidence of interventions that attempt to alter patient–practitioner interactions focus on aspects within the consultation (interpersonal skills and patient/practitioner attributes) without sufficient consideration of the organisational context and how this may impact on patient–practitioner interactions.^10^^,^^11^ Indeed, most previous trials of interventions to alter patient–practitioner interactions have demonstrated small or non-significant effects on outcomes.^17^^,^^18^^,^^49^ These effects may perhaps have been limited by the challenges of the wider organisational context of care, which is not necessarily conducive to optimal interactions. The authors found only one previous trial that considered a ‘whole system’ approach to optimising interactions that took into account all aspects that were of significance to patients within the present study.^50^ This trial on multimorbidity optimised patient–practitioner interactions both within the consultation and in the wider healthcare context, and showed a positive impact on intermediate health outcomes.^50^ The trial included the promotion of longer consultations, relational continuity, in-person time with the practitioner alongside training for practitioners in patient-centred interactions, and self-management support for patients. This multilevel approach to enhancing interactions in multiple morbidity demonstrated a significant impact on healthcare costs and improvements in some domains of patient quality of life. However, to date, no similar studies have been conducted specifically in patients with type 2 diabetes.

### Implications for research and practice

Patient–practitioner interactions are an important component in the delivery of type 2 diabetes care. These interactions have the potential to delay disease progression and associated complications by patient engagement, early diagnosis of risk, and treatment.^10^^,^^13^ Even after an extended period of living with the disease, patients valued sufficient time with, and attention from, their practitioners in order to support them in managing diabetes. This is at odds with current UK and worldwide policy, which often promotes reduced practitioner-personalised care over time. Frequently, personalised encounters with practitioners tend to be more readily available early in the course of a disease with a move towards universal protocols and self-management over time. However, the findings from this study suggest that future efforts to enhance diabetes care may want to consider patient views’ on the importance of the primary care organisational context in facilitating enhanced interactions throughout the course of the disease, which may in turn reduce the risk of complications. This is in contrast to the current NHS policy drive for more efficient and cost-effective health services that is leading to less personalised health care. The patient view has identified the system-wide problems that could limit effective and enhanced patient–practitioner interactions that are not currently being addressed in UK primary care. Further research might consider aspects that could enhance interactions in a more holistic way, through a multilevel system approach rather than interventions that focus on isolated aspects of interactions, which do not consider the healthcare context.
